# Efficacy and Safety of *Ecklonia cava* Kjellman Extract Complex in Respiratory Health: A Randomized, Double‐Blind, Placebo‐Controlled, Multicenter Clinical Trial

**DOI:** 10.1002/fsn3.71449

**Published:** 2026-01-09

**Authors:** Seong‐Cheon Woo, Su Won Lee, Jayun Kim, In Chul Jung, Ji Woong Son, Beom‐Joon Lee, Sang Oh Kwon, Jung‐Hee Byeon, Yang Chun Park

**Affiliations:** ^1^ Division of Respiratory Medicine, Department of Internal Medicine, College of Korean Medicine Daejeon University Daejeon Republic of Korea; ^2^ Department of Neuropsychiatry, College of Korean Medicine Daejeon University Daejeon Republic of Korea; ^3^ Department of Internal Medicine Konyang University Hospital Daejeon Republic of Korea; ^4^ Division of Allergy, Immune and Respiratory System, Department of Internal Medicine, College of Korean Medicine Kyung Hee Korean Medicine Hospital Seoul Republic of Korea; ^5^ R&D Center, S&D Co. Ltd. Cheongju Republic of Korea

**Keywords:** *Chrysanthemum indicum* Linnaeus, cough, dyspnea, *Ecklonia cava* Kjellman, functional food, sputum

## Abstract

Industrialization, air pollution, and respiratory infections have highlighted the importance of maintaining respiratory health. *Ecklonia cava* Kjellman (EC) has anti‐inflammatory, antioxidative, and anti‐allergic effects and therapeutic potential for respiratory symptoms. We evaluated the efficacy and safety of *Ecklonia cava* Kjellman extract complex (EEC), composed of EC and *Chrysanthemum indicum* Linnaeus ( *C. indicum* ) extract, in respiratory health. A randomized, double‐blind, placebo‐controlled, multicenter clinical trial was conducted. Participants (*n* = 106) were randomly allocated to the EEC and placebo groups at a 1:1 ratio and administered EEC or placebo twice daily for 12 weeks, with four visits (screening and weeks 0, 6, and 12). Breathlessness, cough, and sputum scale (BCSS), pulmonary function test (forced vital capacity (FVC), forced expired volume in 1 s (FEV_1_), and FEV_1_/FVC), St. George's respiratory questionnaire (SGRQ), visual analogue scale (VAS), and modified Medical Research Council dyspnea scale (mMRC) were assessed. BCSS (total, breathlessness, and sputum scores), FEV_1_, and VAS (cough and sputum) were significantly improved in the EEC group compared with the placebo group. No significant differences in SGRQ and mMRC between the groups. Safety assessments showed no severe adverse events or clinically significant changes. Given the established pharmacological mechanisms of EC, including its anti‐inflammatory, antioxidative, anti‐allergic, and antimicrobial effects, the results of this study support the potential of EEC for relieving respiratory symptoms and improving respiratory health.

## Graphical abstract

This study evaluated the efficacy and safety of *Ecklonia cava* Kjellman extract complex (EEC) in respiratory health through a randomized clinical trial. Compared to the placebo group, the EEC group showed significant improvements in breathlessness, cough, and sputum scale (BCSS) including total, breathlessness, and sputum scores, visual analogue scale (VAS) for cough and sputum, and FEV_1_, without severe adverse events. These findings will provide evidence supporting the clinical potential of EEC for developing functional foods aimed at alleviating respiratory symptoms.

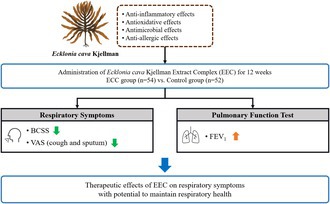

## 1 Introduction

Recently, the incidence of respiratory diseases has increased owing to rapid industrialization and climate change. Urban air pollutants, including particulate matter, ozone, and nitrogen dioxide, have negative effects on allergic respiratory diseases, such as allergic rhinitis and asthma (D'Amato et al. 2013). Westernized lifestyles along with industrialization have led to changes in the residential environment, increased indoor activity, and consumption of processed food, which contribute to an increase in allergic diseases (Ozdemir et al. 2024). Global warming and thunderstorms during the pollen season are correlated with outbreaks of pollen‐induced respiratory allergic diseases (D'Amato et al. 2013; Lindstrom et al. 2017). After the COVID‐19 pandemic, the concerns regarding respiratory infections have increased. The respiratory infections are associated with the development and exacerbation of respiratory diseases, including asthma and chronic obstructive pulmonary disease (COPD) (Busse et al. 2010; Frickmann et al. 2012). Air pollutants also induce oxidative and inflammatory states, making the respiratory tract more susceptible to viral infections such as respiratory syncytial virus (RSV) and influenza (Loaiza‐Ceballos et al. 2022). Considering the increased risk of respiratory diseases, their management, which is directly related to preventing the development and exacerbation of respiratory allergic and infectious diseases, and preparation for the next pandemic have become important.

Respiratory symptoms vary according to the disease and include cough, sputum production, dyspnea, hemoptysis, and chest pain. Although many types of medications, such as antitussives, expectorants, antihistamines, and steroids, have been used to relieve respiratory symptoms, their adverse effects limit their clinical use and make their long‐term use difficult. Codeine, an opioid antitussive that acts on the central nervous system, can cause drowsiness, headaches, constipation, and dyspepsia (Lee et al. 2022). First‐generation antihistamines used to treat cough and allergic diseases can cause anticholinergic side effects, including sedation, constipation, and dry mouth (Malone and Kennedy 2017). Inhaled corticosteroids, which are widely used in patients with asthma, may have local side effects, such as hoarseness, dysphonia, and oral candidiasis, and systemic side effects caused by their absorption into the systemic circulation, such as adrenocortical suppression, osteoporosis, and increased pulmonary infections (N. C. Barnes 2007). Therefore, alternative treatments that are both effective and safe for the treatment of respiratory symptoms are needed.

To provide substitutes for synthetic drugs, recent studies have attempted to develop novel drugs derived from natural products. Researchers have attempted to develop functional foods and dietary supplements for respiratory health and to prove their therapeutic potential, including *Euphorbia hirta* for asthma, *Nigella sativa* (Angiosperm) for asthma and COPD, curcumin for COPD, and *Agrimonia pilosa* (Angiosperm) for influenza (Lim and Mohamed 2016). Marine algae, which contain a variety of bioactive compounds, such as minerals, vitamins, dietary fibers, omega‐3 fatty acids, and carotenoids, have potential nutritional value for industrial and medical purposes (Veluchamy and Palaniswamy 2020). *Ecklonia cava* Kjellman (EC), a brown alga found in coastal areas of Korea and Japan, has long been used as a traditional food and herb. In South Korea, EC is available for industrial utilization, sourced primarily from wild collection in Jeju Island and through controlled cultivation, ensuring a consistent and stable supply of EC (Lee et al. 2025). Many studies have demonstrated that EC has antioxidant, anti‐inflammatory, anti‐allergic, and antimicrobial effects (Wijesinghe and Jeon 2012). EC exerts oxidative effects by controlling radical‐induced damage to the cellular system (Ahn et al. 2007) and the generation of reactive oxygen species (ROS) and nitric oxide (NO) (Kim et al. 2014). EC inhibits the gene expression of MUC5AC mucin in human airway epithelial cells, fine dust‐induced inflammation, and oxidative stress by downregulation of proinflammatory cytokines, including interleukin (IL)‐1β, IL‐6, and tumor necrosis factor (TNF)‐α (Lee and Kwon 2021; Sanjeewa et al. 2020). It also reduces airway inflammation and hyperresponsiveness by inhibiting the T helper type 2 cell response in a mouse model of ovalbumin‐induced asthma (Kim et al. 2008). EC can inhibit neuraminidase on the surface of the influenza virus by enhancing the effects of oseltamivir and viral replication in severe acute respiratory syndrome coronavirus 3C‐like proteinase (SARS‐CoV 3CL^pro^) (Park et al. 2013; Ryu et al. 2011). In addition, it exerts protective effects against particulate matter damage by inhibiting cytotoxicity in nasal and lung epithelial cells and inflammation in mouse models (Park et al. 2019, 2021).

Several clinical trials have investigated its effects on blood glucose and insulin levels in patients with prediabetes (Almutairi et al. 2023; Lee and Jeon 2015) and blood lipid parameters, body fat, and oxidative and inflammatory stress in individuals who are overweight (Lee et al. 2018; Shin et al. 2012). However, no clinical studies have examined the efficacy of EC on respiratory symptoms, including cough, sputum, and dyspnea. In this study, we investigated the *Ecklonia cava* Kjellman extract complex (EEC), composed of EC and *Chrysanthemum indicum* Linnaeus ( *C. indicum* ). *C. indicum* , a plant native to Korea and traditionally used as a medicinal herb, exerts antioxidative, anti‐inflammatory effects, and antimicrobial effects, along with protective effects in acute lung injury and pulmonary fibrosis models (Liang et al. 2025; Nie et al. 2021; Wu et al. 2014). A previous study demonstrated that EEC inhibits NO production and cyclooxygenase (COX)‐2 protein expression by the mitogen‐activated protein kinase (MAPK) signaling pathway, and reduces inflammatory cytokines in an ovalbumin‐induced asthma model (Kang et al. 2023). Considering that inflammation, oxidative stress, allergies, and respiratory infections play important roles in the development and exacerbation of chronic respiratory diseases such as COPD and asthma (P. J. Barnes 2022; Carlier et al. 2021; Jamieson et al. 2013; MacLeod et al. 2021), EEC possesses the potential to relieve respiratory symptoms and maintain a healthy respiratory system. Therefore, this study aimed to demonstrate the efficacy and safety of EEC in respiratory health through a randomized, double‐blind, placebo‐controlled clinical trial.

## 2 Materials and Methods

This trial was conducted in accordance with the Declaration of Helsinki, Korean Good Clinical Practice Guidelines (KGCP), related laws, and protocols. The protocol for this trial was approved by the Institutional Review Board (IRB) of the Daejeon University Daejeon Korean Medicine Hospital (No. DJDSKH‐22‐BM‐09), Konyang University Hospital (No. KYUH IRB 2022‐07‐019), and Kyung Hee Korean Medicine Hospital (No. KOMCIRB IRB 2022‐07‐004). All participants involved in this trial signed an informed consent form. This trial was retrospectively registered on July 17, 2025 at the National Clinical Trial Registry Clinical Research Information Service (https://cris.nih.go.kr) with the identifier number KCT0010785.

### 2.1 Preparation of EEC

EEC is a mixture of EC ethanol extract and *C. indicum* concentrate, which was provided by S&D Co. Ltd. (Cheongju‐si, Chungcheongbuk‐do, Korea). The EC ethanol extract is an individually recognized health‐functional ingredient approved by the Korea Food and Drug Administration (S&D Co. Ltd., Recognition No. 2015‐6). It was purified from the EC ethanol extract and standardized to contain 60 mg/g of dieckol. *C. indicum* was extracted with ethanol, filtered, and concentrated. The EC ethanol extract and the *C. indicum* extract were then blended with dextrin as an excipient, and the mixture was spray‐dried (inlet temperature: 185°C–210°C; outlet temperature: 85°C–98°C) to obtain the EEC powder used in this study. The marker compounds of the EEC were quantified as dieckol (10.8 mg/g) and luteolin‐7‐glucoside (2.2 mg/g) using high‐performance liquid chromatography (HPLC). The entire manufacturing process of the EEC was conducted by S&D Co. Ltd. according to Good Manufacturing Practice (GMP), with its quality control managed under International Organization for Standardization (ISO) certification. The EEC used in this study was produced in the same batch (lot number: SD‐ED‐001).

### 2.2 Study Design

**FIGURE 1:**
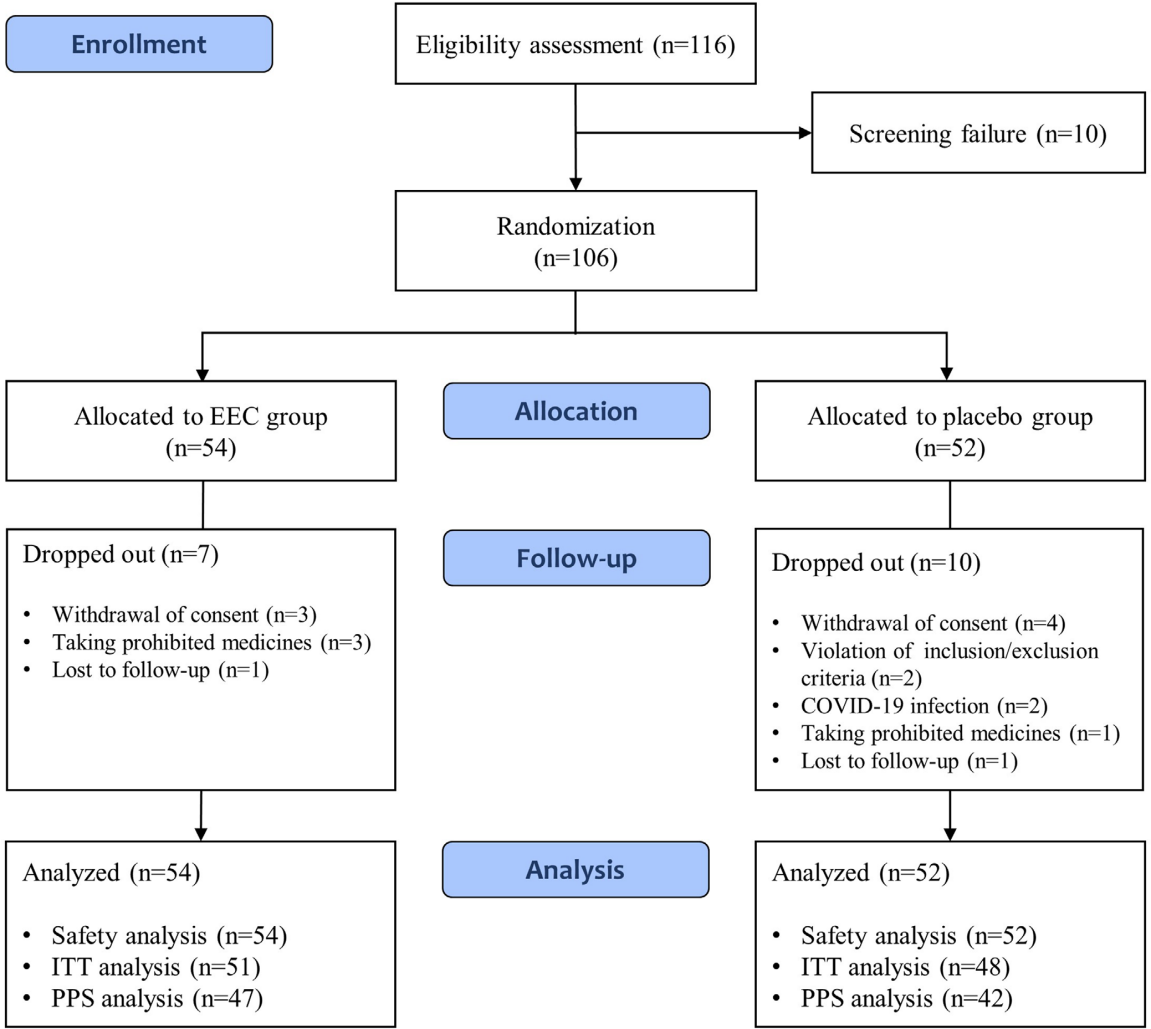
Study flowchart.

This was a 12‐week, randomized, double‐blind, placebo‐controlled, multicenter clinical trial to evaluate the efficacy and safety of EEC in respiratory health. The trial was conducted at three sites (Daejeon University Korean Medicine Hospital, Konyang University Hospital, and Kyung Hee University Korean Medicine Hospital). A total of 106 participants were enrolled from October 28, 2024, to March 18, 2024. Participants who voluntarily agreed to participate in this trial and signed a written informed consent were screened using the inclusion and exclusion criteria. Eligible participants were randomly assigned to either the EEC or control group in a 1:1 allocation ratio. This trial consisted of four scheduled visits (screening visit and weeks 0, 6, and 12). Investigational products were administered twice daily for 12 weeks. The 12‐week treatment duration was established based on prior clinical trials related to EC and respiratory discomforts (Kim et al. 2024; Lee and Jeon 2015; Shin et al. 2012). This period was considered sufficient for the appropriate evaluation of improvements in chronic respiratory symptoms, objective parameters in pulmonary function tests. The detailed study design is illustrated in Figure 1.

### 2.3 Participants

#### 2.3.1 Inclusion Criteria

Participants who met all of the following inclusion criteria were eligible for this trial: (1) males and females aged 19–70 years; (2) more than two symptoms among cough, sputum, and dyspnea (or chest discomfort) lasting at least 1 month and a breathlessness, cough and sputum scale (BCSS) score ≥ 3 points at visit 1; (3) forced expired volume in 1 s (FEV_1_)/forced vital capacity (FVC) ≥ 70% in pulmonary function test; and (4) those who agreed to participate in this clinical trial and signed the written informed consent.

#### 2.3.2 Exclusion Criteria

Those who met any of the following exclusion criteria were excluded from this trial: (1) receiving treatment for severe cardiovascular, immune, gastrointestinal, liver and biliary, renal and urinary, nervous system, musculoskeletal, psychological, or infectious diseases or cancers; (2) clinically significant signs of respiratory diseases on chest radiography; (3) diagnosis of COPD or asthma; (4) chronic bronchitis (BCSS score ≥ 9 points and presence of productive cough and sputum for at least 3 months in 1 year for more than 2 years); (5) respiratory symptoms caused by viral or bacterial infections within 4 weeks at visit 1 (e.g., influenza or COVID‐19^1^); (6) use of adrenocorticoids, immunosuppressants, or antitussive and expectorant medications within 4 weeks at visit 1; (7) consumption of functional foods related to improvement of respiratory health within 2 weeks at visit 1; (8) current smoking or smoking within the past 6 months; (9) average alcohol consumption of > 30 g (210 g/week) per day for males and > 20 g (140 g/week) for females within 4 weeks at visit 1; (10) creatinine levels ≥ 2 times the upper normal limit; (11) aspartate transaminase (AST) and alkaline phosphatase (ALT) levels ≥ 3 times the upper normal limit; (12) unregulated hypertension (systolic blood pressure ≥ 160 mmHg or diastolic blood pressure ≥ 100 mmHg while relaxing for 10 min); (13) unregulated diabetes mellitus (fasting blood sugar level ≥ 180 mg/dL); (14) females who were pregnant, lactating, or planned to become pregnant during the trial; (15) sensitivity or allergy to the ingredients in the investigational products; (16) participation in other clinical trials within 3 months of visit 1 or planned participation in other clinical trials once this trial began; (17) thyroid diseases; and (18) those whom the investigators determined were ineligible to participate in this study.

### 2.4 Interventions

Participants in the EEC and control groups were instructed to take one capsule of EEC or placebo twice daily after meals for 12 weeks. Each 400 mg EEC capsule comprised EEC (350 mg), microcrystalline cellulose (34 mg), silicon dioxide (8 mg), and magnesium stearate (8 mg). Each 400 mg placebo capsule comprised maltodextrin (330 mg), caramel coloring (20 mg), microcrystalline cellulose (34 mg), silicon dioxide (8 mg), and magnesium stearate (8 mg). Investigational products (EEC and placebo) were produced by S&D Co. (Cheongju, Republic of Korea).

### 2.5 Randomization and Blinding

Eligible participants were allocated to the EEC and placebo groups in a 1:1 ratio by blocked randomization at visit 2. A randomization list was generated before the clinical trial using SAS version 9.4 statistical software (SAS Institute Inc., Cary, NC, USA). The manufacturer of the investigational products labeled the identification codes of the participants according to the randomization list and provided them to the institutions before the clinical trial. To maintain blinding, the investigators managed the allocation of identification codes, which were sealed and remained undisclosed, except for emergencies, during which the identification code should be identified. The investigators provided investigational products consistent with the randomization numbers assigned to the participants. If the investigational products were lost or damaged, spare products were provided while maintaining blinding.

### 2.6 Outcome Measures

#### 2.6.1 Breathlessness, Cough, and Sputum Scale

The BCSS is a self‐rating 5‐point Likert scale to evaluate common respiratory symptoms, including breathlessness, cough, and sputum, and has reliability and validity for the symptom severity in patients with COPD (Leidy, Schmier, et al. 2003). The BCSS was assessed at visits 1, 3, and 4.

#### 2.6.2 Pulmonary Function Testing

The FVC and FEV_1_ were measured using pulmonary function tests. FVC is the total volume of air exhaled during a rapid forced exhalation after maximal inspiration. The FEV_1_ is the maximum volume of air expelled in 1 s in a forced capacity test. FEV_1_/FVC, which indicates the severity of the airflow limitation, was also measured (Swanney et al. 2008). Pulmonary function tests were conducted at visits 1 and 4.

#### 2.6.3 St. George's Respiratory Questionnaire

The St. George's Respiratory Questionnaire (SGRQ) is a self‐administered questionnaire developed for asthma and COPD (Jones et al. 1991). It comprised 50 items with three components: symptoms (distress due to respiratory symptoms), activity (disturbance of daily activities), and impact (psychosocial function). The symptoms, activity, impact, and total scores were measured at visits 1, 3, and 4.

#### 2.6.4 Visual Analogue Scale

The Visual Analogue Scale (VAS) was used as a subjective rating scale to evaluate cough, sputum, and dyspnea (or chest discomfort). A 100 mm horizontal VAS was used, in which 0 mm means “no symptom” and 100 mm means “unbearable symptom.” The VAS scores for cough, sputum, and dyspnea were evaluated at visits 1, 3, and 4.

#### 2.6.5 Modified Medical Research Council Dyspnea Scale

The modified Medical Research Council Dyspnea Scale (mMRC) is a 5‐point scale (0–4) based on the severity of dyspnea that is used to classify patients with COPD (Bestall et al. 1999). The mMRC grades were as follows: grade 0, breathlessness only with strenuous exercise; grade 1, breathlessness when hurrying on the level or walking up a slight hill; grade 2, walking slower than other people of the same age on the level due to breathlessness or the need to stop for breath when walking at their own pace; grade 3, shortness of breath after walking a few minutes on the level or about 100 yards; and grade 4, too breathless to leave the house or breathless when dressing or undressing. The mMRC was assessed at visits 2, 3, and 4.

### 2.7 Sample Size

The sample size calculation was based on the results of a previous study (Kirichenko et al. 2020) using the BCSS score as the primary outcome measure. With reference to the previous study, we estimated the mean difference in BCSS scores between the study groups as 0.655, with a standard deviation of 1.00. Adopting a significance level (*α*) of 5% and a power (1 − *β*) of 80%, the calculated sample size was 37 participants per group.

The sample size in each group was calculated using the following equation:

(1) $$\left\{\frac{2{\left(Z_{\frac{\alpha }{2}}+Z_{\beta }\right)}^{2}\times \sigma^{2}}{{∆}^{2}}\right\}=\left\{\frac{2{\left(1.96+0.84\right)}^{2}\times 1.00^{2}}{{\left(-0.655\right)}^{2}}\right\}\approx 37$$

Considering a 30% dropout rate, 53 participants per group (106 total participants) were required.

### 2.8 Statistical Analysis

Data analysis was conducted using SAS version 9.4 statistical software (SAS Institute Inc., Cary, NC, USA). Analysis of the full analysis set (FAS) was performed based on intention‐to‐treat (ITT) analysis of all randomized participants. The FAS analysis group was defined as participants who took the investigational products at least once and underwent at least one efficacy evaluation without violating the inclusion/exclusion criteria. The per‐protocol set (PPS) analysis group was defined as the participants who completed the trial without serious violations that could affect the results of the FAS group. Data on efficacy were analyzed using PPS analysis, as the main analysis, and FAS analysis additionally. Demographic analyses were performed using PPS analysis, and safety analysis was performed using the Safety Set analysis, in which all participants were randomized and administered the investigational products at least once. Data were described as the mean ± standard deviation (SD), and the significance testing of differences followed a two‐sided *p*‐value < 0.05. The two‐sample *t*‐test or Wilcoxon rank‐sum test was used, depending on their normality. Within‐group comparisons were performed using paired *t*‐test. In the demographic analysis, continuous variables were analyzed using a two‐sample *t*‐test or Wilcoxon rank‐sum test, depending on their normality. Categorical variables are expressed as frequency and rate, and the *χ* ^2^ test or Fisher's exact test was performed. If the between‐group differences in the demographic and lifestyle analyses were clinically significant, a generalized linear model (GLM) was conducted, with the underlying characteristics as covariates in the efficacy analysis. Safety analysis was performed to evaluate adverse events (AEs), laboratory test results (complete blood test, blood chemistry test, and urine analysis), vital signs (blood pressure and pulse rate), and body weight.

## 3 Results

### 3.1 Study Participants

Initially, 116 participants were screened, and 10 who did not meet the inclusion criteria were excluded. The remaining 106 participants were randomly allocated to the EEC (*n* = 54) or placebo (*n* = 52) group. Four participants in the EEC group dropped out owing to withdrawal of consent and loss to follow‐up. Eight participants in the placebo group dropped out owing to withdrawal of consent, violation of the inclusion/exclusion criteria, loss to follow‐up, or administration of prohibited medicines. Finally, 94 participants completed the study (EEC group, *n* = 50; placebo group, *n* = 44). A CONSORT flowchart of this study is shown in Figure 1.

Five participants dropped out without an efficacy evaluation after visit 2, and two participants dropped out because of violations of the inclusion/exclusion criteria after visit 3. Therefore, 99 participants were included in the FAS analysis. In the FAS analysis, four participants dropped out after visit 3, and five participants were excluded owing to COVID‐19 infection and the administration of prohibited medicines after completion of this trial. In total, 89 participants were included in the PPS analysis (EEC group, *n* = 47; placebo group, *n* = 42).

### 3.2 Demographic and Baseline Characteristics

Table 1 shows the baseline characteristics of the 89 participants included in the PPS analysis. There were no differences in sex, height, body weight, fertility, occupation, type of symptoms (cough, sputum, and dyspnea), ratios of smoking and alcohol consumption, and exposure time to the atmospheric environment. However, the age was significantly different between the EEC and placebo group (34.06 ± 10.25 vs. 29.83 ± 8.95 years; *p* = 0.0344), which was adjusted in the analysis (Table 1).

**TABLE 1:** Baseline characteristics.

Characteristic	EEC group (*n* = 47)	Placebo group (*n* = 42)	*p*
Age, mean ± SD, year	34.06 ± 10.25	29.83 ± 8.95	0.0344^a^
Sex, *n* (%)			0.8163^b^
Male	19 (40.43)	18 (42.86)	
Female	28 (59.57)	24 (57.14)	
Presence of fertility, *n*	24	23	0.3577^c^
Height, mean ± SD, cm	166.51 ± 7.97	167.36 ± 8.39	0.6240^d^
Weight, mean ± SD, kg	64.94 ± 13.50	66.44 ± 14.46	0.6811^a^
Smoking experience, *n* (%)	10 (21.28)	7 (16.67)	0.5807^b^
Smoker (current), *n* (%)	0 (0.00)	0 (0.00)	—
Smoking cessation, *n* (%)	10 (100.00)	7 (100.00)	—
Smoking cessation over 6 months, *n* (%)	10 (100.00)	7 (100.00)	—
Drinker (current), *n* (%)	19 (40.43)	18 (42.86)	0.8163^b^
Exposure time to atmospheric environment, mean ± SD, h/day	3.29 ± 2.88	3.67 ± 3.01	0.2422^a^
Symptoms, *n* (%)			
Cough	37 (78.72)	36 (85.71)	0.3912^b^
Sputum	43 (91.49)	37 (88.10)	0.7299^c^
Dyspnea (or chest discomfort)	19 (40.43)	14 (33.33)	0.4892^b^
None	0 (0.00)	0 (0.00)	—

^a^ *p*‐value is derived from the Wilcoxon rank sum test.

^b^ *p*‐value is derived from Chi‐square test.

^c^ *p*‐value is derived from Fisher's exact test.

^d^ *p*‐value is derived from two sample *t*‐test.

### 3.3 BCSS

The BCSS total scores in the EEC and placebo groups decreased by 2.49 ± 2.38 and 1.64 ± 2.52, respectively, after 6 weeks and by 3.38 ± 2.28 and 2.02 ± 3.27, respectively, after 12 weeks. The changes from baseline to 6 weeks were not significantly different between the groups (*p* = 0.1030); however, the total scores from baseline to 12 weeks significantly decreased in the EEC group, compared with those in the placebo group (*p* = 0.0208).

**FIGURE 2:**
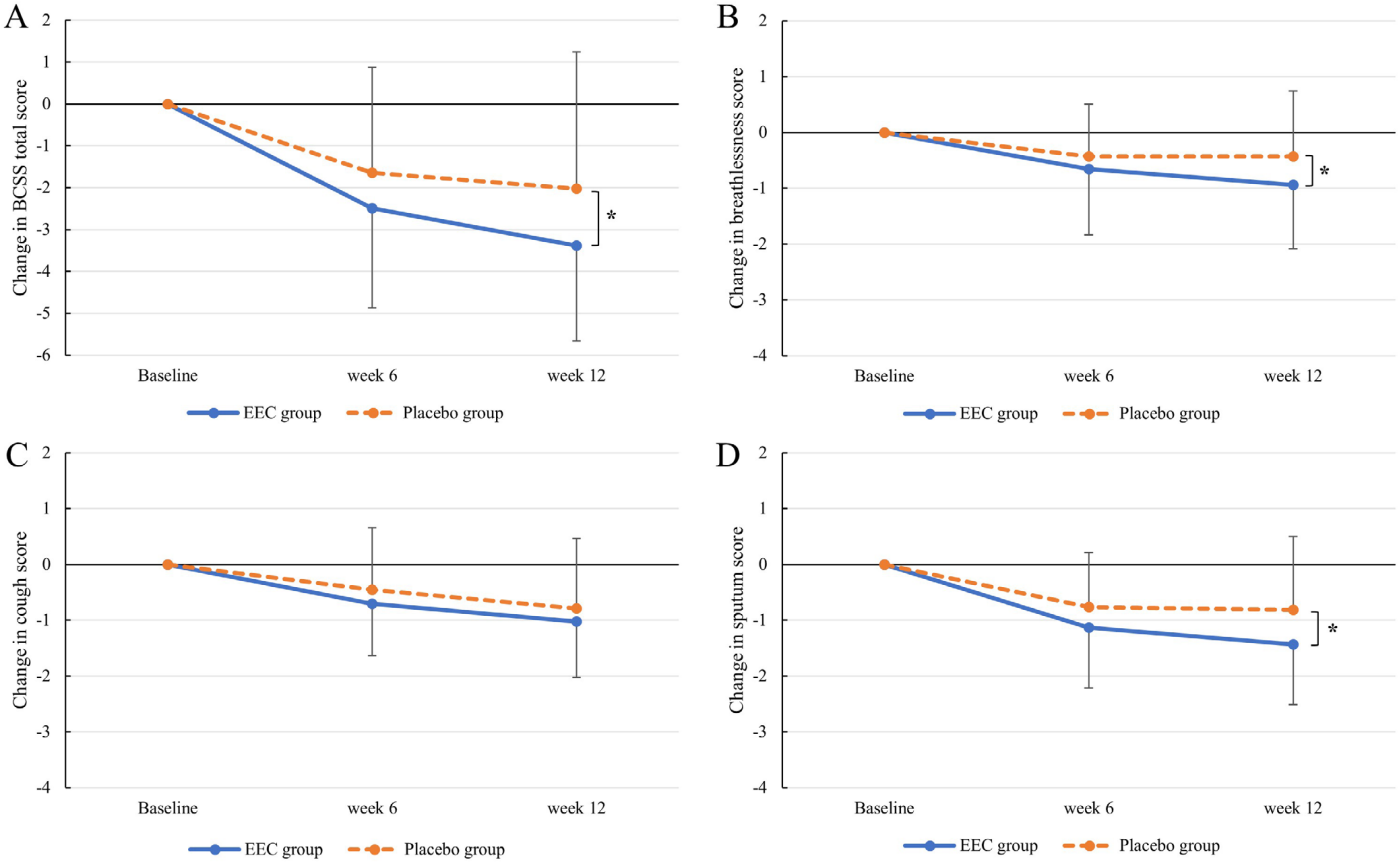
Changes in BCSS scores from baseline to weeks 6 and 12. BCSS (A) total, (B) breathlessness, (C) cough, and (D) sputum scores. *p < 0.05; significant difference between the EEC and placebo groups.

The dyspnea scores decreased in the EEC and placebo groups by 0.66 ± 1.18 and 0.43 ± 0.94, respectively, after 6 weeks and by 0.94 ± 1.15 and 0.43 ± 1.17, respectively, after 12 weeks. The changes from baseline to 6 weeks were not significantly different between the groups (*p* = 0.2783); however, the dyspnea scores significantly decreased from baseline to 12 weeks in the EEC group, compared with those in the placebo group (*p* = 0.0395). The cough scores in the EEC and placebo groups decreased by 0.70 ± 0.93 and 0.45 ± 1.11, respectively, after 6 weeks and by 1.02 ± 1.01 and 0.79 ± 1.26, respectively, after 12 weeks. The changes from baseline to 6 and 12 weeks were not significantly different (*p* = 0.2060 and 0.3015, respectively). The sputum scores in the EEC and placebo groups decreased by 1.13 ± 1.08 and 0.76 ± 0.98, respectively, after 6 weeks and by 1.43 ± 1.08 and 0.81 ± 1.31, respectively, after 12 weeks. The changes from baseline to 6 weeks were not significantly different (*p* = 0.1346); however, the changes from baseline to 12 weeks were significantly different (*p* = 0.0148) (Figure 2, Table 2).

**TABLE 2:** Breathlessness, cough, and sputum scale (BCSS) scores in the EEC and placebo groups at baseline (BL) and after 6 and 12 weeks.

PP set	EEC group (*n* = 47)			Placebo group (*n* = 42)			*p* ^b^	
	Baseline	Week 6	Week 12	Baseline	Week 6	Week 12	W6‐BL	W12‐BL
Total scores	4.98 ± 1.73	2.49 ± 1.76	1.60 ± 1.69	4.62 ± 1.55	2.98 ± 2.43	2.60 ± 2.79	0.1030	0.0208^†^
Change from BL		−2.49 ± 2.38	−3.38 ± 2.28		−1.64 ± 2.52	−2.02 ± 3.27		
*p* ^a^		< 0.0001*	< 0.0001*		0.0001*	0.0003*		
Breathlessness scores	1.43 ± 1.04	0.77 ± 0.73	0.49 ± 0.62	1.14 ± 0.68	0.71 ± 0.81	0.71 ± 0.97	0.2783	0.0395^†^
Change from BL		−0.66 ± 1.18	−0.94 ± 1.15		−0.43 ± 0.94	−0.43 ± 1.17		
*p* ^a^		0.0004*	< 0.0001*		0.0052*	0.0225*		
Cough scores	1.60 ± 0.80	0.89 ± 0.73	0.57 ± 0.65	1.67 ± 0.72	1.21 ± 1.00	0.88 ± 1.09	0.2060	0.3015
Change from BL		−0.70 ± 0.93	−1.02 ± 1.01		−0.45 ± 1.11	−0.79 ± 1.26		
*p* ^a^		< 0.0001*	< 0.0001*		0.0116*	0.0002*		
Sputum scores	1.96 ± 0.95	0.83 ± 0.79	0.53 ± 0.75	1.81 ± 0.97	1.05 ± 1.01	1.00 ± 1.13	0.1346	0.0148^†^
Change from BL		−1.13 ± 1.08	−1.43 ± 1.08		−0.76 ± 0.98	−0.81 ± 1.31		
*p* ^a^		< 0.0001*	< 0.0001*		< 0.0001*	0.0003*		

^a^ *p*‐value is derived from the Paired *t*‐test to compare within group (*p < 0.05).

^b^ *p*‐value is derived from GLM (ANCOVA) adjusted age to compare between the EEC and placebo group (^†^ *p* < 0.05).

### 3.4 Pulmonary Function Test

The changes in the FVC from baseline to 12 weeks were not significantly different between the groups (*p* = 0.1755). The FVC decreased by 0.02 ± 0.46 and 0.12 ± 0.32 L in the EEC and placebo groups, respectively, after 12 weeks. The changes in the FEV_1_ from baseline to 12 weeks were significantly different between the groups (*p* = 0.0256). The FEV_1_ decreased by 0.01 ± 0.36 and 0.17 ± 0.38 L in the EEC and placebo groups, respectively, after 12 weeks. The changes in the FEV_1_/FVC from baseline to 12 weeks were not significantly different between the groups (*p* = 0.1477). The FEV_1_/FVC increased by 0.15% ± 3.50% in the EEC group and decreased by 1.71% ± 8.06% in the placebo group, after 12 weeks (Table 3).

**TABLE 3:** Pulmonary function test results in the EEC and placebo groups at baseline (BL) and after 12 weeks.

PP set	EEC group (*n* = 47)		Placebo group (*n* = 42)		*p* ^b^
	Baseline	Week 12	Baseline	Week 12	W12‐BL
FVC (L)	4.07 ± 1.11	4.05 ± 1.04	4.11 ± 1.00	3.99 ± 0.98	0.1755
Change from BL		−0.02 ± 0.46		−0.12 ± 0.32	
*p* ^a^		0.7233		0.0159*	
FEV_1_ (L)	3.42 ± 0.86	3.42 ± 0.84	3.47 ± 0.81	3.29 ± 0.79	0.0256^†^
Change from BL		−0.01 ± 0.36		−0.17 ± 0.38	
*p* ^a^		0.8767		0.0050*	
FEV_1_/FVC (%)	84.60 ± 5.93	84.74 ± 5.98	84.71 ± 5.94	83.00 ± 8.88	0.1477
Change from BL		0.15 ± 3.50		−1.71 ± 8.06	
*p* ^a^		0.7719		0.1758	

^a^ *p*‐value is derived from the Paired *t*‐test to compare within group (*p < 0.05).

^b^ *p*‐value is derived from GLM (ANCOVA) adjusted age to compare between the EEC and placebo group (^†^ *p* < 0.05).

### 3.5 SGRQ

The changes in the SGRQ total, symptom, activity, and impact scores from baseline to 6 and 12 weeks were significantly decreased within the groups. However, no significant differences were observed between the groups from baseline to 6 or 12 weeks (Table 4).

**TABLE 4:** St. George's respiratory questionnaire (SGRQ) scores in the EEC and placebo groups at baseline (BL) and after 6 and 12 weeks.

PP set	EEC group (*n* = 47)			Placebo group (*n* = 42)			*p* ^b^	
	Baseline	Week 6	Week 12	Baseline	Week 6	Week 12	W6‐BL	W12‐BL
Total scores	26.56 ± 14.63	16.57 ± 13.51	11.90 ± 11.80	25.95 ± 17.09	17.37 ± 14.19	13.45 ± 14.07	0.7745	0.5561
Change from BL		−9.99 ± 12.39	−14.65 ± 14.23		−8.58 ± 14.64	−12.51 ± 18.60		
*p* ^a^		< 0.0001*	< 0.0001*		0.0005*	< 0.0001*		
Symptoms	43.73 ± 16.72	29.45 ± 16.20	22.04 ± 15.73	41.97 ± 16.41	30.12 ± 14.62	24.91 ± 14.83	0.6432	0.3025
Change from BL		−14.28 ± 19.05	−21.70 ± 21.09		−11.85 ± 17.14	−17.07 ± 19.81		
*p* ^a^		< 0.0001*	< 0.0001*		< 0.0001*	< 0.0001*		
Activity	34.15 ± 21.68	23.03 ± 20.80	15.79 ± 19.06	31.57 ± 20.69	24.67 ± 20.05	18.90 ± 20.86	0.3214	0.1821
Change from BL		−11.12 ± 16.43	−18.36 ± 19.32		−6.90 ± 17.60	−12.67 ± 22.59		
*p* ^a^		< 0.0001*	< 0.0001*		0.0150*	0.0008*		
Impacts	15.29 ± 13.57	7.63 ± 10.63	5.64 ± 8.58	16.41 ± 19.04	7.91 ± 13.65	5.70 ± 12.28	0.6691	0.6984
Change from BL		−7.66 ± 12.27	−9.65 ± 12.54		−8.50 ± 17.75	−10.72 ± 20.51		
*p* ^a^		< 0.0001*	< 0.0001*		0.0035*	0.0016*		

^a^ *p*‐value is derived from the Paired *t*‐test to compare within group (*p < 0.05).

^b^ *p*‐value is derived from GLM (ANCOVA) adjusted age to compare between the EEC and placebo group.

### 3.6 VAS

The VAS for cough decreased in the EEC and placebo groups by 21.19 ± 26.39 and 9.24 ± 28.06 mm, respectively, after 6 weeks and by 28.79 ± 27.78 and 13.07 ± 29.67 mm, respectively, after 12 weeks; significant differences were observed between groups from baseline to 6 and 12 weeks (*p* = 0.0442 and 0.0096, respectively). The VAS for sputum decreased in the EEC and placebo groups by 27.53 ± 26.45 and 11.95 ± 27.29 mm, respectively, after 6 weeks and by 34.83 ± 28.99 and 16.60 ± 34.61 mm, respectively, after 12 weeks; significant differences were observed between groups from baseline to 6 and 12 weeks (*p* = 0.0094 and 0.0140, respectively). The VAS for dyspnea (or chest discomfort) decreased in the EEC and placebo groups by 19.36 ± 27.45 and 7.31 ± 30.77 mm, respectively, after 6 weeks and by 25.64 ± 28.67 and 13.05 ± 30.51 mm, respectively, after 12 weeks; no significant differences were observed between groups from baseline to 6 or 12 weeks (*p* = 0.0789 and 0.0591, respectively) (Table 5).

**TABLE 5:** Visual analogue scale (VAS) scores in the EEC and placebo groups at baseline (BL) and after 6 and 12 weeks.

PP set	EEC group (*n* = 47)			Placebo group (*n* = 42)			*p* ^b^	
	Baseline	Week 6	Week 12	Baseline	Week 6	Week 12	W6‐BL	W12‐BL
Cough (mm)	38.30 ± 25.64	17.11 ± 16.11	9.51 ± 12.85	32.81 ± 21.73	23.57 ± 24.69	19.74 ± 25.37	0.0442^†^	0.0096^†^
Change from BL		−21.19 ± 26.39	−28.79 ± 27.78		−9.24 ± 28.06	−13.07 ± 29.67		
*p* ^a^		< 0.0001*	< 0.0001*		0.0389*	0.0067*		
Sputum (mm)	48.60 ± 28.72	21.06 ± 21.38	13.77 ± 18.68	44.57 ± 30.55	32.62 ± 30.13	27.98 ± 29.14	0.0229^†^	0.0140^†^
Change from BL		−27.53 ± 26.45	−34.83 ± 28.99		−11.95 ± 27.29	−16.60 ± 34.61		
*p* ^a^		< 0.0001*	< 0.0001*		0.0070*	0.0034*		
Dyspnea (or chest discomfort) (mm)	35.79 ± 29.79	16.43 ± 18.73	10.15 ± 15.51	28.12 ± 24.48	20.81 ± 24.09	15.07 ± 23.86	0.0789	0.0591
Change from BL		−19.36 ± 27.45	−25.64 ± 28.67		−7.31 ± 30.77	−13.05 ± 30.51		
*p* ^a^		< 0.0001*	< 0.0001*		0.1314	0.0083*		

^a^ *p*‐value is derived from the Paired *t*‐test to compare within group (*p < 0.05).

^b^ *p*‐value is derived from GLM (ANCOVA) adjusted age to compare between the EEC and placebo group (^†^ *p* < 0.05).

### 3.7 mMRC

The changes in mMRC grades from baseline to both 6 and 12 weeks significantly decreased within the groups; however, no significant differences were observed between the groups from baseline to 6 or 12 weeks (*p* = 0.1702 and 0.0687, respectively). mMRC grades decreased in the EEC and placebo groups by 0.57 ± 1.28 and 0.26 ± 0.63, respectively, after 6 weeks and by 0.83 ± 1.22 and 0.38 ± 0.96, respectively, after 12 weeks (Table 6).

**TABLE 6:** Modified medical research council dyspnea scale (mMRC) scores in the EEC and placebo groups at baseline (BL) and after 6 and 12 weeks.

PP set	EEC group (*n* = 47)			Placebo group (*n* = 42)			*p* ^b^	
	Baseline	Week 6	Week 12	Baseline	Week 6	Week 12	W6‐BL	W12‐BL
mMRC	1.26 ± 1.03	0.68 ± 0.73	0.43 ± 0.62	0.93 ± 0.68	0.67 ± 0.53	0.55 ± 0.86	0.1702	0.0687
Change from BL		−0.57 ± 1.28	−0.83 ± 1.22		−0.26 ± 0.63	−0.38 ± 0.96		
*p* ^a^		0.0035*	< 0.0001*		0.0099*	0.0140*		

^a^ *p*‐value is derived from the Paired *t*‐test to compare within group (*p < 0.05).

^b^ *p*‐value is derived from GLM (ANCOVA) adjusted age to compare between the EEC and placebo group.

### 3.8 Safety Assessment

Safety assessments were performed using the Safety Set, which included participants who took the investigational products at least once after randomization. In total, 106 participants (EEC group, *n* = 54; placebo group, *n* = 52) were included in the safety assessment.

There were 19 AEs in 16 participants in the EEC group (29.63%) and 20 AEs in 13 participants in the placebo group (25.00%), with no significant between‐group differences (*p* = 0.5930). No severe AEs were reported, and no participants dropped out due to AEs. AEs were considered “mild” and “definitely not related to the product” (*n* = 14) and “probably not related to the product” (*n* = 25) by investigators. Common AEs in the EEC group were gastrointestinal and respiratory disorders, such as dyspepsia and pharyngitis. Common AEs in the placebo group were respiratory disorders such as pharyngitis. Other AEs, including back pain, headache, skeletal pain, dysmenorrhea, dermatitis, and conjunctivitis, were also reported.

No significant between‐group differences in vital signs or body weights were observed. In laboratory examinations, blood urea nitrogen (BUN) and leukocyte levels in urine analysis were significantly different between groups from baseline to 12 weeks (*p* = 0.0084 and 0.0299, respectively), which were not considered clinically significant by investigators.

## 4 Discussion

In the primary outcome analysis, the total BCSS score in the EEC group decreased significantly by 3.38 ± 2.28 points from baseline to Week 12. This change surpassed −1.3 points representing a highly effective treatment, suggesting that EEC effectively alleviates the respiratory symptom (Leidy, Rennard, et al. 2003). The BCSS and VAS scores for all three symptoms (cough, sputum, and dyspnea) in the placebo group significantly decreased from baseline to 12 weeks. The effects of the placebo may have two explanations. First, participants who had respiratory symptoms for more than 1 month without chronic respiratory diseases such as COPD, asthma, and chronic bronchitis were included in this trial; thus, their symptoms may not be chronic and severe or could be cured spontaneously without treatment. Although they had respiratory symptoms, the symptoms were mild; the baseline BCSS total scores were 4.98 ± 1.73 in the EEC group and 4.62 ± 1.55 in the placebo group, out of 12 possible points, which may reflect their clinical effects on respiratory symptoms. The objective of this study is not to demonstrate the curative effect for severe respiratory diseases such as those targeted by prescribed medications, but to demonstrate the therapeutic effect of EEC as a functional food to relieve mild respiratory symptoms in individuals without explicit respiratory diseases. Consequently, these results are meaningful for alleviating the mild respiratory symptoms, including cough, sputum, and dyspnea. Second, the placebo effects may have affected the evaluations. Physiological effects related to the stimulation of salivation and mucus secretions due to taste and psychological effects through expectation, conditioning, reward, and anxiety reduction can evoke the placebo effects of cough medicines (Eccles 2020). Several studies have demonstrated that placebo interventions reduce bronchoconstriction in patients with asthma, which is mediated by the enhanced vagal activation of the lungs (Meissner 2014; Wolters et al. 2019). Given that the BCSS, SGRQ, VAS, and mMRC are subjective self‐rating tools, the participants may be susceptible to the placebo effect when rating the severity of their respiratory symptoms. However, we also utilized pulmonary function tests for objective evaluation, which would reflect the effects of EEC more clearly than subjective evaluations. To evaluate more reliable efficacy on respiratory symptoms, methodological limitations in the study design, production of the placebo, and outcome measures require improvements.

In the pulmonary function test, while the between‐group differences in the changes in FEV_1_ were significant from baseline to 12 weeks, the changes in FVC and FEV_1_/FVC were not significantly different between the groups. The FVC, FEV_1_, and FEV_1_/FVC in the EEC group did not significantly change. Participants with normal lung function were included in this trial, which limited the verification of significant effects in the pulmonary function test. However, the FEV_1_ is widely used in the diagnosis and prognosis of COPD and for monitoring the response to treatments (Doherty 2008), and the effects on the FEV_1_ may indicate the possibility of improving pulmonary function. In addition, those without a reduced FEV_1_/FVC can experience symptoms such as dyspnea, cough, and sputum and have lung dysfunction with risks for the development of COPD, suggesting that managing their symptoms and the potential risks of COPD is necessary (Han et al. 2021).

In the safety assessment, no significant differences were observed in the incidence of AEs between the groups. No severe AEs were observed, and no participants dropped out due to AEs during this trial. In addition, no clinically significant changes in vital signs, body weight, or laboratory parameters were observed, indicating the safety of EEC.

This study has some limitations. The included participants had mild respiratory symptoms without impaired pulmonary function or chronic respiratory diseases that would affect the results of this trial, and no significant between‐group differences in changes in the FVC, FEV_1_/FVC, SGRQ scores, and mMRC grades from baseline to 6 and 12 weeks were observed. Although we could not evaluate the efficacy for patients with respiratory diseases, these results are meaningful as the objective of this study was to demonstrate the efficacy of EEC for individuals with mild respiratory symptoms. Furthermore, more participants are required to demonstrate the efficacy of EEC. We calculated the sample size based on a previous study (Kirichenko et al. 2020) that used the BCSS score as an outcome measure, applied other investigational products, and recruited participants with COPD who were only male and smokers (> 10 packs/years) with reduced FEV_1_ and FEV_1_/FVC, which was different from the participants included in this study. Despite these limitations, the randomized, double‐blinded, placebo‐controlled clinical trial design to evaluate the efficacy of EEC ensures high internal validity of the results. Furthermore, we observed statistically significant results in both the subjective outcome measure (BCSS) and the objective physiological parameter (FEV1). These improvements support that EEC possesses significant therapeutic effects on respiratory symptoms. Further studies should consider these limitations and explore better study designs to assess the validity and reliability of the efficacy results.

## 5 Conclusions

In conclusion, this study demonstrated the therapeutic effects of EEC as functional foods for individuals experiencing mild respiratory symptoms. EEC administration resulted in statistically significant improvements in both subjective symptoms, including cough, sputum, and dyspnea, and the objective physiological parameter of FEV1 in pulmonary function tests. EEC administration was confirmed to be safe, without any clinically significant AEs. These findings will provide evidence supporting the clinical potential of EEC for developing functional foods aimed at alleviating respiratory symptoms. Further studies are needed to elucidate the mechanisms underlying the therapeutic effects on respiratory symptoms and to evaluate the efficacy of EEC through well‐designed clinical trials.

## Author Contributions

**Seong‐Cheon Woo:** writing – original draft, visualization, investigation. **Su Won Lee:** investigation. **Jayun Kim:** investigation. **In Chul Jung:** writing – review and editing. **Ji Woong Son:** resources, investigation. **Beom‐Joon Lee:** resources, investigation. **Sang Oh Kwon:** project administration, funding acquisition. **Jung‐Hee Byeon:** project administration, investigation. **Yang Chun Park:** writing – review and editing, supervision.

## Funding

This research was supported by grants from S&D Co. Ltd.; the National Research Foundation of Korea, funded by the Ministry of Science and ICT (grant number: RS‐2022‐NR069365); and the Korea Health Technology R&D Project through the Korea Health Industry Development Institute, funded by the Ministry of Health & Welfare (grant number: RS‐2023‐KH139099).

## Ethics Statement

The protocol for this trial was approved by the Institutional Review Board (IRB) of the Daejeon University Daejeon Korean Medicine Hospital (No. DJDSKH‐22‐BM‐09), Konyang University Hospital (No. KYUH IRB 2022‐07‐019), and Kyung Hee Korean Medicine Hospital (No. KOMCIRB IRB 2022‐07‐004). All participants involved in this trial signed an informed consent form.

## Conflicts of Interest

The authors declare no conflicts of interest.

## Data Availability

Data will be made available on request.

## Endnotes

^1^
The existing respiratory symptoms had deteriorated or new respiratory symptoms developed due to COVID‐19. Those without additional respiratory symptoms due to COVID‐19 could participate in this trial at four weeks after release from quarantine. Those with additional respiratory symptoms could participate in this trial at four weeks after there were no differences compared to the status before the COVID‐19 infection.
